# Decitabine-induced kidney thrombotic microangiopathy with glomerular crescents formation and tubular necrosis: A case report

**DOI:** 10.1097/MD.0000000000022901

**Published:** 2020-10-23

**Authors:** Ai-bo Qin, Ying Tan, Tao Su

**Affiliations:** Renal Division, Department of Medicine, Peking University First Hospital; Institute of Nephrology, Peking University; Key Laboratory of Renal Disease, Ministry of Health of China; Key Laboratory of CKD Prevention and Treatment, Ministry of Education of China, Beijing, China.

**Keywords:** decitabine, thrombotic microangiopathy, crescent formation, tubular necrosis

## Abstract

**Introduction:**

Chemotherapeutic agents of direct cell damage play a role in initiating thrombotic microangiopathy (TMA), however still being underdiagnosed. Decitabine (DAC) is a pyrimidine analogue of the nucleoside cytidine, which can lead to injury to endothelium. Biopsy-proven DAC-induced kidney injury is rare.

**Patient concerns:**

A 47-year-old Chinese man with membranous nephropathy presented recurrent edema and acute kidney injury after a 3-day course of low dose DAC infusion because of cyclophosphamide-relating thrombocytopenia.

**Diagnosis:**

Laboratory data revealed nephrotic syndrome, hematuria, renal glycosuria and hypokalemia with hyperchloridemia. Renal pathological findings revealed TMA with secondary glomerular crescents formation (28%), partial foot process effacement and acute tubular necrosis. A diagnosis of DAC-induced renal TMA was considered.

**Interventions:**

As DAC had been timely discontinued before admission, the patient only received supportive treatment.

**Outcomes:**

The patient achieved rapid remission of acute kidney injury after DAC withdrawal, and his serum creatinine further decreased to normal level after 6 months.

**Conclusion:**

Careful monitoring of renal function especially serum creatinine should be emphasized during DAC treatment.

## 1 Introduction

Thrombotic microangiopathy (TMA) is a group of critical syndromes with specific pathologic features showing arteriolar or capillary damage, characterized by microvascular thrombosis occlusion, microangiopathic hemolytic anemia, thrombocytopenia and target-organ dysfunction (ie, acute kidney injury, [AKI]). Among various secondary causes, drug-induced etiology is an important one, which is considered to play a pathogenetic role in initiating TMA. There are many drugs that are associated with TMA, such as chemotherapy drugs, quinine, cyclosporine, tacrolimus, and antibiotics.^[1]^ These identified chemotherapy drugs include mitomycin C, gemcitabine, platinum salts and pegylated liposomal doxorubicin.^[2]^ However, there is sometimes neither thrombocytopenia nor hemolytic anemia in kidney TMA. This may not be proved until kidney biopsies are performed. Glomerular crescents formation happens to be observed in TMA cases.^[3]^

Decitabine (5-aza 2′-deoxycytidine, DAC) is a pyrimidine analogue of the nucleoside cytidine.^[4]^ As a potent DNA demethylating agent, DAC produces cytotoxicity by inhibiting DNA methylation in rapidly dividing cells and has effect to control myelodysplastic syndromes, acute or chronic myelogenous leukemia.^[5–9]^ At low doses, DAC could promote megakaryocyte maturation and platelet (PLT) production in immune thrombocytopenia.^[10]^ Clinical trials have reported good tolerance. Herein, we report a rare case of kidney TMA with crescents and tubular necrosis induced by DAC in a preceding membranous nephropathy (MN) patient.

## 2 Case presentation

A 47-year-old man complained of aggravated edema for 10 days’ duration. He had a history of nephrotic syndrome for almost 2 years by supportive therapy until 4 months ago when he was diagnosed with typical MN at a local hospital. The patient was administered oral prednisone 60 mg/d initially combined with intravenous cyclophosphamide (CTX) 0.8 g every 2 weeks until 6 weeks ago when he happened to notice petechiae on his body. The treatment of CTX was discontinued (the accumulated dose of CTX was nearly 5.0 g). Although no alternative agent was utilized after the discontinuation of CTX, his urine protein decreased from a baseline 12.7 g/d to 2.7 g/d and he achieved partial remission. On examination, his blood pressure was 140/85 mmHg. Laboratory data showed normal white blood cell and hemoglobin, the PLT count was 60 × 10^3^/μL and it dropped to 10 × 10^3^/μL 2 weeks later, but the hemoglobin level remained at 12.2 g/dl. Impaired megakaryocyte maturation and PLT production were observed in bone marrow smear. The patient was suspected of immune thrombocytopenic purpura. He undertook once PLT transfusion and was injected a low-dose DAC at 10 mg per day for 3 days. No diarrhea occurred. However, the patient complained recurrent edema with an almost 15 kg weight gain the following 10 days, meanwhile, his serum creatinine (SCr) level was elevated from a baseline 89 μmol/L to 392 μmol/L, but the PLT count returned to 69 × 10^3^/μL (Fig. 2).

The patient was admitted for investigation and treatment to Peking University First Hospital. On admission, the physical examination revealed a blood pressure of 168/105 mmHg. He had a “moon face” and severe anasarca. Laboratory data collected at the time of admission showed SCr level was spontaneously declined to 256 μmol/L, with an increase of PLT count to 194 × 10^3^/μL. Serum LDH was 460 IU/mL, Coombs’ test was negative and no schistocytes were detected. Urine protein was 11.9 g/d, sediments red blood cells were 8-10/HP. Anti-phospholipase A2 receptor (PLA2R) antibody was 107 RU/mL (<20 RU/mL). Complement 3 (C3), C4, and factor H concentrations were in normal range. Anti-factor H, anti-glomerular basement membrane (GBM) and anti-neutrophil cytoplasmic antibodies were negative. No monoclonal immunoglobulin was detected from serum and urine by immune fixation electrophoresis. As shown in Table 1, hypokalemia with hyperchloremia, hypouricemia and renal glycosuria (serum glucose was 4.75 mmol/L) suggested tubular injury. No sign of renal venous thrombosis was found by ultrasound examination. Taken together, a diagnosis of new-onset AKI with hematuria after DAC exposure, superimposed on a pre-existing but non-remission MN could be made. The occurrence of severe thrombocytopenia is considered CTX-relating adverse reaction, because thrombocytopenia happened 4 weeks prior to the increase of SCr, and started recovery around 4 weeks after CTX withdrawal (Fig. 2).

**Table 1:**
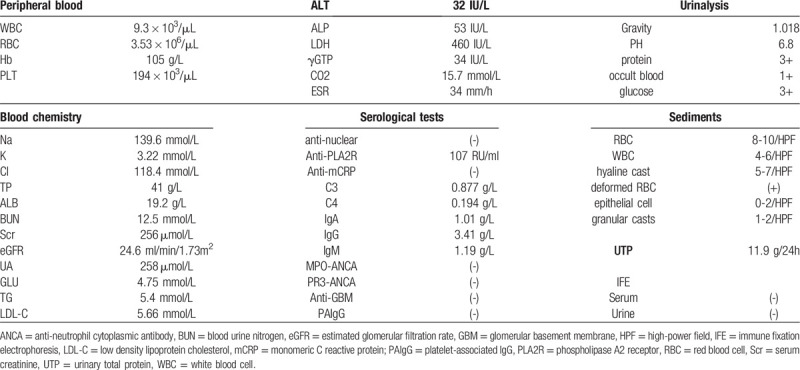
Laboratory data on admission.

**Figure 1:**
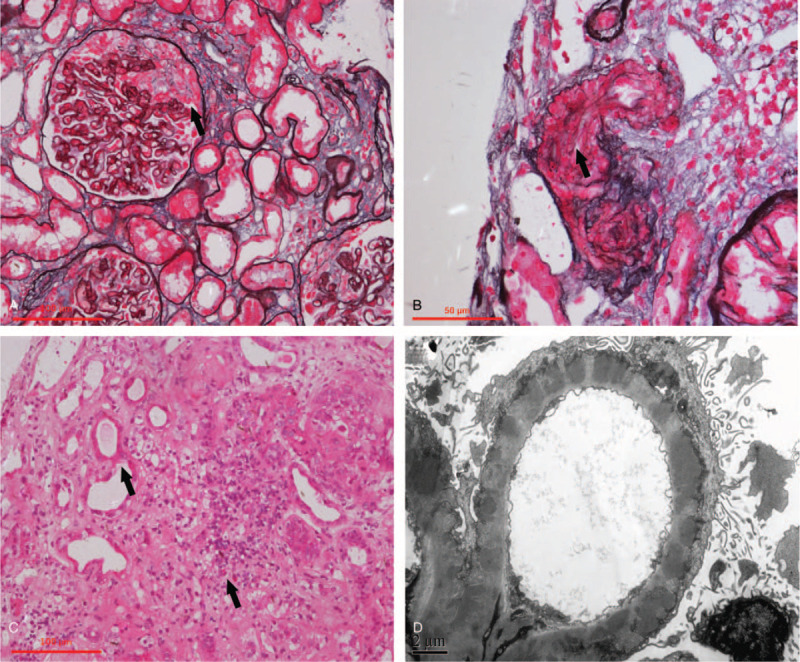
Findings on Kidney biopsy. (A) A glomerulus showing extracapillary cellular crescent on the basis of diffusely thickened glomerular basement membrane (PASM + Masson staining, ×200). (B) One involved arteriole showing obvious expansion of subendothelial zone and myxoid edema with narrowed vascular lumen (PASM + Masson staining, ×400). (C) Vacuolization and granular degeneration of tubular epithelial cells, naked renal tubules, and mildly infiltrated lymphoplasmacytes in the interstitium (Hematoxylin-Eosin staining, ×200). (D) A large amount of electron density deposits and foot process effacement (Electron microscopy, ×8000).

The patient underwent a repeat renal biopsy. Biopsy sample included 50 glomeruli and 7 of them showed ischaemic sclerosis. All the glomeruli demonstrated an MN pattern as diffuse GBM thickening with subepithelial immune complex deposits, and foot process effacement (Fig. 1A and D). Twelve glomeruli (28% of total) showed involvement by crescents, including 6 cellular and 6 fibro-cellular crescents (Fig. 1A). No fibrinoid necrosis was found. Immunofluorescence staining revealed granular-like deposit along the GBM as IgG (3+), C3 (3+), PLA2R (2+), IgA (-), IgM (-). One arteriole showed obvious segmental expansion of subendothelial zone and myxoid edema with narrowed vascular lumen, but glomerular capillaries remained intact (Fig. 1B). Tubular epithelial cells presented severe vacuolization and granular degeneration, several naked renal tubules with detached epithelial cell fragments, and mildly infiltrated lymphoplasmacytes in the interstitium (Fig. 1C). No dense deposit was observed in mesangial region under electronic microscope.

**Figure 2:**
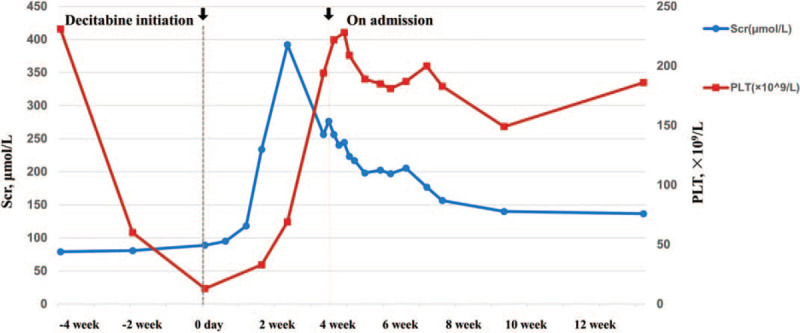
The change of serum creatinine and platelet count during the course of the disease. PLT = platelet; Scr = serum creatinine.

As DAC had already been discontinued before admission, the patient's renal function got a further recovery (Fig. 2). Three weeks later when he was dismissed, the SCr was 157 μmol/L. And the SCr was finally maintained at 115 to 134 μmol/L the following 6 months. The patient was given another course of prednisone and oral CTX at 25 mg/d for the treatment of MN. No thrombocytopenia relapsed. The final diagnosis was DAC-induced TMA with glomerular crescents and tubular necrosis superimposed on pre-existing anti-PLA2R related MN.

## 3 Discussion

TMA is a disease with special histopathological characteristics involving glomeruli and/or arterial endocapillary cell proliferation and swelling. It could be triggered by drugs, infections and systemic diseases. Typical presentation is the presence of microangiopathic hemolysis anemia, thrombocytopenic purpura, renal and neurological abnormalities. However, diagnosis of TMA may sometimes be delayed or misdiagnosed, because drug-induced bone marrow depression is usually firstly suspected. Sometimes in case of kidney TMA, thrombocytopenia and hemolytic anemia can be transient or even absent. The progressive kidney failure, new or exacerbated hypertension are valuable clues.^[2]^ Vascular damage manifested by arteriolar or glomerular capillary thrombosis with endothelium and vessel wall injuries are definite pathological findings of TMA. Here we reported a case of renal TMA with crescents formation and tubular necrosis induced by DAC. Despite of no obvious presentation of systemic TMA, pathological findings provided more helpful information. In the absence of common causes leading to TMA such as diarrhea and infection, the DAC was identified the culprit. Toxic effect of DAC on renal tubular epithelial cells also contributed to the occurrence of AKI.

Chemotherapeutic agents serve as one major cause of TMA but still being underdiagnosed. Vascular injury showing TMA as a result is reported with increasing frequency, because all the chemo-therapeutic drugs have direct cell damage to endothelial cells. DAC is a deoxycytidine analogue as an antineoplastic agent. Its antineoplastic efficacy comes from 2 distinct mechanisms: cytotoxicity and induction of hypomethylation. DAC was approved in 2006 for high-risk myelodysplastic syndromes of all French-American-British subtypes.^[5]^ In recent years, the treatment of immune thrombocytopenia with low-dose of DAC has been studied in some centers.^[10]^ Nephrotoxicity of DAC is mild, rare and dose-dependent overall. In phase II clinical trials, DAC can lead to direct injury to endothelium. There have been 3 clinical trials describing an increase of serum creatinine in several patients (3/21, 5/50 and 1/66, respectively).^[7–9]^ Only 1 trial detailed the information of DAC associated kidney injury in 3 elder patients.^[7]^ The SCr was 97-115 μmol/L before DAC infusion, and increased by 41.7%, 63.6%, 46.2% respectively within 3 weeks after therapy at the dose of 30, 75, 100 mg/m^2^. Among them, the renal function of 1 patient improved rapidly 1 week after drug withdrawal, while for another patient who was treated with a second course of DAC resulting in a further increase of SCr, and a delayed recovery to 4 months after drug withdrawal. This patient was finally reported a biopsy-proven acute tubulointerstitial nephritis. Here in the current study, we reported a special case of DAC associated AKI as renal TMA, crescents formation and tubular necrosis, however the remission of renal function after withdrawal was relatively satisfactory. At present, most of the DAC-based chemotherapy regimens are given every 3-6 weeks. And as described above, mild kidney injury recovered quickly, so it is reasonable to measure SCr within 1 week after DAC infusion and prior to the start of next chemotherapy. In patients with underlying kidney disease, Scr and urine test should both be monitored.

The prevalence of crescents formation in MN is rare. In a cohort published recently, 12 of these 15 patients diagnosed with MN and necrotizing crescentic glomerulonephritis, were associated with anti-neutrophil cytoplasmic antibody and anti-GBM antibodies.^[11]^ Rodriguez EF et al reported that in all antibodies-negative MN patients, average 25% (range, 2%-73%) were involvement with crescents.^[12]^ Crescents could also be induced by TMA. The reported prevalence is around 5.6%, average 26% of glomeruli. The frequency is similar as that we reported in the current patient. Crescent formation was speculated to be induced by endocapillary hypercellularity in response to severe injury to the glomerular capillary or arteriole wall, indicating worse long-term prognosis.

It is well-known that there is almost 50% of TMA patients with genes mutations coding different components of complement cascade. Patients carrying specific human leukocyte antigen (HLA) alleles such as *HLA-DRB1∗11* were associated with TMA and affecting clinical outcome.^[13]^ MN is also recognized as a disease having genetic predisposition. HLA alleles *DRB1 ∗1501 DRB1 ∗0301* are independent risk alleles, and *DRB1 ∗1502* indicates worse renal outcome of MN.^[14]^ Thus, it is supposed that toxic drugs act as the initiating event of TMA in patients predisposed by genetic defects in the complement cascade with high-risk HLA alleles.

To conclude, we reported a case of DAC-induced TMA with crescents formation and tubular necrosis. The patient achieved rapid remission of TMA after immediate discontinuation of the offending drug. It is worthy of careful monitoring of renal function during DAC treatment.

## Author Contributions

**Approval of final manuscript:** all authors.

**Conceptualization:** Tao Su and Ying Tan.

**Data curation:** Ai-bo Qin.

**Validation:** Tao Su

**Writing – original draft preparation:** Ai-bo Qin.

**Writing – review & editing:** Tao Su and Ying Tan.
